# Regional Differentiation of Ground–Active Beetle Assemblages Across Contrasting Dryland Alfalfa Agroecosystems

**DOI:** 10.3390/insects17101030

**Published:** 2026-10-08

**Authors:** Qi He, Yingjie Luo, Xueqin Liu, Zhaozhi Lu, Shujuan Chen, Jian Wang

**Affiliations:** 1 School of Forestry and Grassland Science, Ningxia University, Yinchuan 750021, China; heqi_1820@126.com (Q.H.); yingjieluo010434@163.com (Y.L.); 2 Institute of Crop Science, Ningxia Academy of Agriculture and Forestry Sciences, Yinchuan 750021, China; wjzwssd@163.com; 3 College of Energy and Chemical Engineering, Ningxia Vocational and Technical University of Industry and Commerce, Yinchuan 750021, China; 4 College of Plant Health and Medicine, Qingdao Agriculture University, Qingdao 266109, China; zhaozhi_lv@sina.com; 5 Institute of Assets Inventory, Ningxia Department of Natural Resources, Yinchuan 750002, China; chen-85@hotmail.com

**Keywords:** activity density, arid agroecosystems, beta diversity, environmental filtering, *Medicago sativa*, trophic guilds, Carabidae, Scarabaeidae, Tenebrionidae

## Abstract

Perennial crops can provide a shared habitat template for arthropods, but whether regional differentiation persists among fields of the same crop in drylands remains unclear. From 2020 to 2022, we surveyed ground–active beetles at 55 perennial alfalfa sites across mountainous, oasis, and desert regions of Ningxia, China. We compared beetle diversity and feeding–guild structure among regions and examined their relationships with climate, soil, and vegetation. A total of 48,093 individuals representing 105 species, 62 genera, and 10 families were recorded. Agroecological region explained 30.1% of compositional variation and was associated with differences in total assemblages and all guilds. Mountainous sites had higher overall species richness and were dominated by detritivores; oasis sites contained a greater proportion of herbivores despite similar herbivore activity density among regions, whereas desert sites were numerically dominated by carabids and predators but had lower richness and diversity within the predatory guild. Regional differentiation was more evident in richness, assemblage composition, and trophic organization than in Shannon and Simpson diversity. β–diversity was dominated by nestedness. Climate was associated mainly with activity density and alpha diversity, whereas vegetation showed more recurrent associations with beta diversity. Ground–active beetle assemblages therefore remained regionally distinct despite occurring in the same perennial crop, supporting biodiversity assessment that accounts for dryland regional context.

## Simple Summary

Alfalfa fields share a perennial crop, but they occur under strongly contrasting dryland conditions. We surveyed ground–active beetles in 55 alfalfa sites distributed among mountainous, irrigated–oasis, and desert–edge regions of Ningxia, China. The three regions supported distinct taxonomic and feeding–guild assemblages. Mountainous sites contained the largest species pool and were dominated by detritivores. Oasis sites had a greater proportional contribution of herbivores, although their herbivore activity density was not higher. Desert–edge sites were dominated numerically by carabids and predators but supported fewer predatory species and lower predator diversity. Regional differences were clearer for species richness, assemblage composition, feeding–guild organization, and beta diversity components than for whole–assemblage Shannon or Simpson diversity. Climate was associated mainly with activity density and alpha diversity, whereas vegetation showed more recurrent associations with beta diversity. These findings show that a common crop does not create ecologically equivalent habitats across dryland regions. Biodiversity assessment and management of alfalfa agroecosystems should therefore account for regional environmental context and should not rely on a single taxonomic group or diversity metric.

## 1. Introduction

Dryland farmland biodiversity reflects the combined influence of agricultural management and pronounced regional environmental heterogeneity [1,2,3,4]. Even under the same crop, spatial variation in water availability, temperature, soil properties, and vegetation structure can impose contrasting environmental filters on local communities [5,6,7]. These filters may have distinct effects on activity density, species richness, assemblage composition, and β–diversity [8,9]. Taxa and feeding guilds differ in resource use, trophic position, and tolerance to environmental stress. Their responses to regional environmental gradients are therefore unlikely to be congruent [10,11,12,13]. Yet most previous studies have focused on single taxonomic groups or trophic levels and a limited set of diversity metrics. Whether taxonomic and trophic components exhibit parallel patterns across contrasting agroecological regions remains unresolved [14]. Integrating multiple community dimensions within a shared crop context is therefore necessary to clarify associations between regional environmental heterogeneity and variation in dryland farmland biodiversity. This integration can provide a stronger basis for region–specific conservation and management [12,15,16].

Alfalfa fields are important perennial agroecosystems in dryland regions. Compared with annual croplands, alfalfa fields have longer production cycles and more continuous vegetation cover, which can provide arthropods with persistent habitat and food resources [17,18]. However, mowing periodically disrupts this apparent stability by altering canopy structure, litter availability, microclimatic conditions, and resource distribution during the growing season [17,19]. Alfalfa is also cultivated across dryland regions that can differ substantially in hydrothermal conditions, soil properties, and vegetation structure [5,6]. Growing the same crop therefore represents a shared habitat template but does not imply ecological equivalence among regions. Together, crop continuity and regional environmental variation make alfalfa fields a useful system for evaluating whether regional differentiation remains evident and, if so, in which community dimensions.

Ground–active beetles are particularly informative for this evaluation because of their taxonomic diversity, varied trophic positions, and sensitivity to environmental variation. They also contribute to litter decomposition, soil processes, herbivory, and biological control [14,20,21,22]. Beetle families and feeding guilds differ in resource dependence, trophic position, habitat requirements, and tolerance to environmental stress. These differences may lead to contrasting responses [23,24,25,26]. Previous studies, primarily on carabids, indicate that soil fertility, mowing, crop resources, and landscape context can affect activity density, dominant species, and trait composition, even when overall species richness or diversity changes little. For example, Wang et al. (2022) [24] found that non–predatory carabid richness and activity density declined with increasing soil nitrogen in apple orchards on the North China Plain. In these orchards, the response of predatory carabids to mowing varied with surrounding landscape complexity [24]. Deppe et al. (2023) found no significant differences in overall carabid richness or activity density between traditional and simplified modern agricultural landscapes in Europe [25]. Regional climate may also explain substantial variation in assemblage composition beyond that associated with local resources and disturbance [8,27]. These findings suggest that aggregate α–diversity metrics alone may fail to reveal important changes in taxonomic composition and trophic organization [25,27]. However, whether the total ground–active beetle assemblage, dominant beetle families, and different feeding guilds exhibit congruent regional patterns within perennial alfalfa systems remains unclear. It is also unclear whether regional β–diversity primarily reflects species replacement or nestedness. How climatic, soil, and vegetation conditions are associated with different community dimensions also remains unresolved.

We therefore surveyed ground–active beetle assemblages at 55 perennial alfalfa sites distributed across the mountainous, oasis, and desert regions of Ningxia, China, from 2020 to 2022. We addressed four questions: (1) Do beetle activity density, α–diversity, and assemblage composition differ among the three regions despite the cultivation of the same crop? (2) Are regional response patterns congruent among the total assemblage, dominant families, and predatory, herbivorous, omnivorous, and detritivore guilds? (3) Does regional β–diversity primarily reflect species replacement or nestedness? (4) How are climatic, soil, and vegetation conditions associated with activity density, α–diversity, β–diversity, and assemblage composition, including potential indirect coefficients? We expected regional responses to differ among dominant families and feeding guilds because of differences in their resource requirements and environmental tolerances. Finally, we also predicted that climatic conditions would be associated with beetle communities both directly and indirectly, with associations occurring through local soil and vegetation characteristics. This study assesses regional differentiation in dryland alfalfa agroecosystems and provides a basis for region–specific biodiversity management.

## 2. Materials and Methods

### 2.1. Study Areas

The study is situated in the Ningxia Hui Autonomous Region, China, where alfalfa occupies 4.88 million mu (approximately 325,000 hectares). The region provides a suitable setting for comparing biodiversity among oasis, desert, and mountainous agroecosystems. The mountainous region encompassed Longde and Pengyang counties in Guyuan City (35°21′–36°17′ N, 105°48′–106°58′ E). This region has a temperate continental climate ranging from subhumid to semiarid, with a mean elevation of 1888 m, a mean annual temperature of 5.6 °C, and mean annual precipitation of 470 mm. The oasis region was located within the Yellow River oasis belt and included Hongsibu District and Yinchuan City (37°25′–38°52′ N, 106°03′–106°53′ E). This region has a temperate continental climate, with a mean elevation of 1302 m, a mean annual temperature of 8.8 °C, and mean annual precipitation of 226 mm. The desert region was located in Pingluo County (37°29′–39°25′ N, 105°48′–106°52′ E). It has a continental climate, with a mean elevation of 1098 m, a mean annual temperature of 8.9 °C, and mean annual precipitation of 133 mm. Alfalfa cultivation has been widely promoted in Ningxia since 2000. Alfalfa management practices are similar across oasis, desert, and mountainous regions and include irrigation and fertilization through the growing season.

### 2.2. Experimental Design

**Figure 1:**
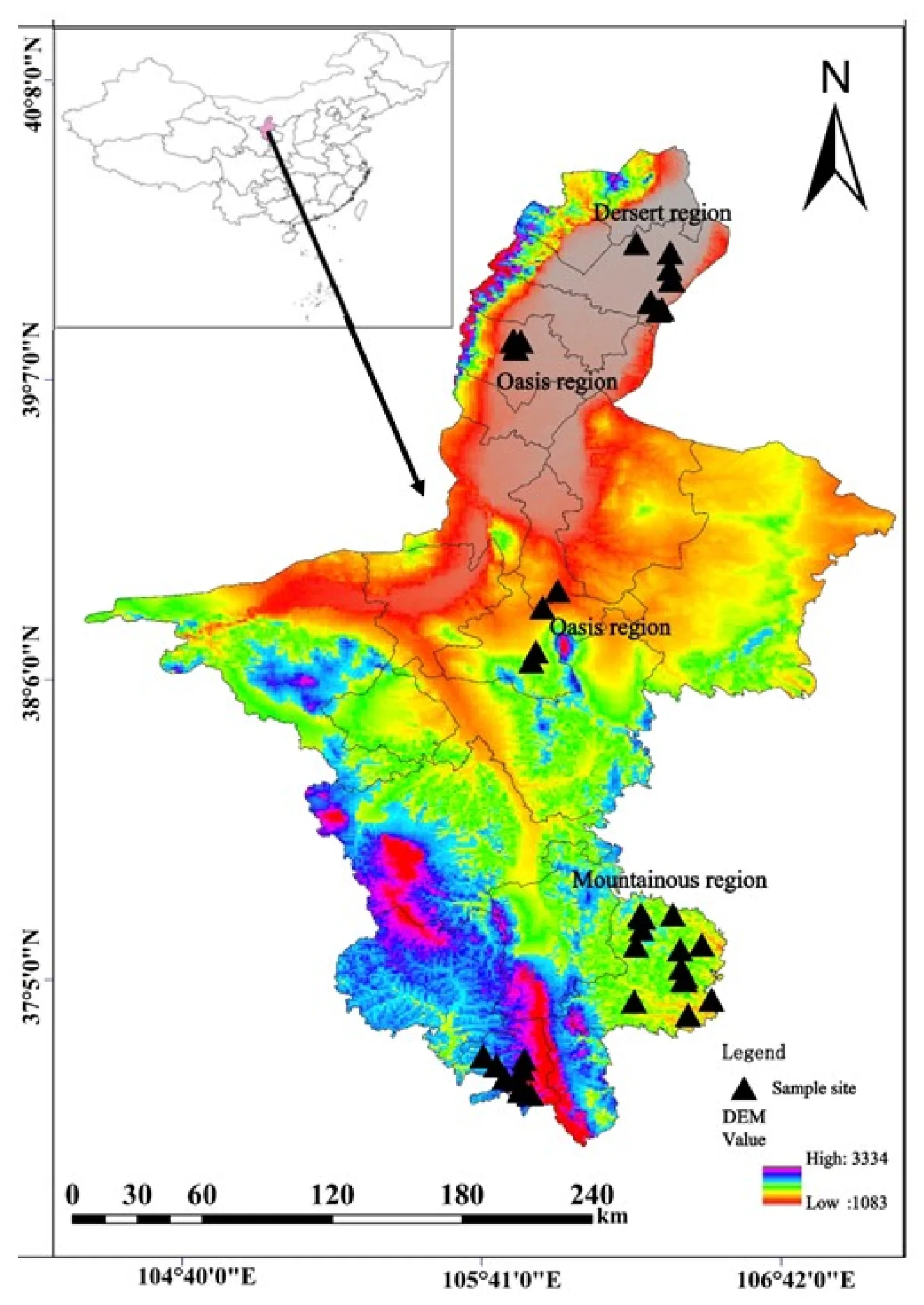
Study area and sampling sites.

We used a hierarchical repeated–measures design across three agroecological regions of Ningxia. A total of 55 sites were selected, including 34 sites in the mountainous region, 9 in the oasis region, and 12 in the desert region. At each site, we randomly selected three perennial alfalfa fields as spatial replicates, yielding 165 fields in total. Monthly sampling was conducted at 165 sample sites from May 2020 to September 2022. Alfalfa had been cultivated in these fields for the past five years, and sampling locations remained fixed across years. Ground-active beetles, vegetation and soil were during monthly sampling round. The complete sampling design comprised 2475 field–by–sampling–round observations. The study sites are shown in Figure 1.

### 2.3. Insect Sampling

Ground–active beetles were sampled using pitfall traps once per month from May to September in each of the three survey years [28,29,30]. During each monthly sampling round, five traps were placed in a five–point arrangement within each 20 m × 20 m plot, with adjacent traps at least 5 m apart. Each trap consisted of a plastic cup with an opening diameter of 7.15 cm and a depth of 9 cm. The cup was buried with its rim level with the soil surface and contained a mixture of vinegar, sugar, ethanol, and water at a volume ratio of 2:1:1:20. The traps remained active for three consecutive days during each sampling round. Captures from the five traps within a field were pooled to produce one field–level species–abundance sample for each monthly sampling round. For descriptive presentation, activity density was expressed as the number of individuals per trap per three–day sampling period. For descriptive summaries, activity density was expressed as the number of individuals per trap per three day sampled period. In count models, the pooled integer catch from the five traps served as the response variable rather than the mean catch per trap. When sampling effort differed because of trap loss or damage, the effective number of trap–days was recorded and used to account for sampling effort through an offset in the count model [30]. All traps were removed from the field after each sampling round to avoid environmental contamination. Collected specimens were preserved in 75% ethanol and transported to the laboratory (Sinopharm Chemical Reagent Co., Ltd., Shanghai, China). Beetles were identified to family, genus, and species based on morphological characteristics examined under a stereomicroscope (SZM–168, Motic Asia, Hong Kong, China). Individuals of each species were counted. Species were classified into four feeding guilds—herbivores, predators, omnivores, and detritivores—according to their documented diets [31,32,33,34]. When species–level dietary information was unavailable or uncertain, species were assigned to guilds based on the predominant diet reported for their genus or family. Guild assignments used dietary information relevant to the sampled life stage; supporting sources are listed in Table S6 [35,36,37,38]. Table S6 also identifies guild assignments inferred from genus– or family–level information. Guild assignments remain uncertain when diets vary with life stage or resource availability.

### 2.4. Environment Measurements

Vegetation was measured concurrently with every monthly beetle survey to ensure temporal alignment between the two datasets. During each sampling round, one 0.5 m × 0.5 m vegetation quadrat was established within each 20 m × 20 m field plot for vegetation assessment and soil sampling. Herbaceous vegetation cover (P.Cv, %) was estimated visually, and plant density (P.Dn, plant/m^2^), plant height (P.Ht, cm), and species richness (P.SR, number of species) were recorded. All aboveground plant material within the quadrat was then harvested, placed in paper bags, oven–dried at 70 °C, and weighed to determine aboveground herbaceous biomass (P.Bm, g/m^2^). Surface litter was also collected and weighed to determine litter mass (SL, g/m^2^). During each sampling round, three soil subsamples were collected from the 0–10 cm soil layer within the vegetation quadrat. The three subsamples were pooled into a single composite sample for each field during each sampling round. Each composite sample was sealed in a plastic bag and transported to the laboratory for air–drying and sieving. Soil temperature (ST, °C), soil compaction (SC, g/cm^−3^) and soil water content (SWC, %) were measured using soil water potential and temperature Meter (TRS–II–300, Zhejiang Top Cloud–agri Technology Co., Ltd., Hangzhou, China), soil pH was measured in situ per site using a YSI multiparameter water quality meter (YSI 6600, YSI Inc., Yellow Springs, OH, USA), total carbon (TC, g/kg) was measured using dichromate oxidation external heating method, total nitrogen (TN, g/kg) was measured using the ammonium molybdate method (EPA 300.0). Total phosphorus (TP, g/kg) was analyzed by persulfate digestion (EPA 365.3). Elevation (Alt), latitude (Lat), and longitude (Long) were recorded using a handheld GPS receiver (eTrex 30x, Garmin International, Inc., Olathe, KS, USA). Mean annual temperature (MAT), mean annual relative humidity (MAH), and mean annual precipitation (MAP) were obtained from local meteorological stations.

### 2.5. Statistical Analyses

Ground–active beetle assemblages collected from five traps within each site were pooled for analysis. Species relative abundance was calculated across the three agroecological regions, and the species were classified as dominant (>10%), common (1–10%), or rare (<1%). Activity density, expressed as the number of ground–dwelling arthropods captured per trap per three–day sampling period, was calculated along with species richness, Shannon–Wiener index, and Simpson index. Regional differences in univariate community metrics were analyzed using generalized linear mixed models (GLMM) [39,40]. Agroecological region was included as the focal fixed effect, with a random intercept for sampling site to account for between–site heterogeneity and repeated dependence among observations from the same site. Models were initially fitted using a Poisson error distribution and a log–link function. When simulation–based residual diagnostics indicated overdispersion, the models were refitted with a negative binomial distribution. If effective trapping effort differed among samples, the logarithm of effective number of trap–days was included as an offset [40]. Observed species richness, equivalent to Hill diversity of order *q* = 0, was analyzed using a count model. Hill diversities of orders *q* = 1 and *q* = 2, corresponding respectively to the exponential of Shannon entropy and the inverse of Simpson concentration, were analyzed. These metrics were analyzed using Gaussian or alternative continuous–response models, with model suitability assessed through residual distributions [41,42,43]. The overall effect of region was evaluated using likelihood–ratio tests. When the overall regional effects were significant, estimated marginal means were compared pairwise using Tukey adjustment for multiple testing [36]. To account for the large number of generalized linear mixed models (GLMMs) reported in Tables S1–S5, all *p* values were adjusted for multiple comparisons using the Benjamini–Hochberg false discovery rate (FDR) procedure. Corrections were applied within each table (i.e., across the 56 GLMM tests in Tables S1, S2, S4 and S5, and across the 24 PERMANOVA pairwise comparisons in Table S3), and adjusted *q* values are reported alongside the raw *p* values. Effects were considered significant at FDR *q* < 0.05; marginally non–significant effects (0.01 < *p* < 0.05 that did not survive correction) are described as trends. Model performance was assessed using simulation–based residual diagnostics for overdispersion, zero inflation, outliers, and deviations from residual uniformity [44].

Regional differences in assemblage composition were analyzed using the site–level species matrix containing one effort–standardized assemblage vector for each of the 55 sites. Species activity density values were square–root transformed to reduce the influence of highly abundant species, and Bray–Curtis dissimilarities were calculated from the transformed data. Regional differences in assemblage composition were visualized using non–metric multidimensional scaling (NMDS) and tested using PERMANOVA with 9999 permutations [45,46,47]. The number of NMDS dimensions, final stress, number of random starts, and convergence were reported for every assemblage component. The homogeneity of multivariate dispersion among regions was evaluated using the same site set and Bray–Curtis dissimilarity matrix. Distances from sites to their regional centroid were calculated using the betadisper function. Regional differences in dispersion were tested using PERMDISP with 9999 permutations [48,49]. When the overall PERMDISP test was significant, pairwise comparisons were conducted with *p* values adjusted for multiple testing. *p* values from pairwise PERMANOVA comparisons among regions were also adjusted using the Holm procedure. PERMANOVA results were interpreted alongside Permdisp results to assess whether regional differences could reflect variation in multivariate dispersion as well as centroid location.

β–diversity was analyzed using the same site–level assemblage data used for NMDS and PERMANOVA. Species activity density data were converted to presence–absence data, and total Sørensen dissimilarity was partitioned into species–turnover and nestedness–resultant components using the betapart package [50,51]. Total beta diversity was measured by Sørensen dissimilarity between sampling occasions and partitioned into turnover and nestedness components. Because sample sizes differed among the mountainous, irrigated–oasis, and desert–edge regions, the robustness of β–diversity estimates was evaluated using balanced resampling. In each of 999 iterations, nine sites were randomly selected without replacement from each region, matching the smallest regional sample size. Multisite total β–diversity, turnover, and nestedness–resulant components were recalculated in each iteration [52]. Balanced resampling was applied exclusively for β–diversity partitioning. GLMM and PERMANOVA were used only on the full 55–site dataset, without balanced subsampling. These methods can accommodate unequal regional sample sizes.

To identify key environmental correlates of ground–active beetle communities, we first examined bivariate relationships using Spearman rank correlations and evaluated predictor importance via random forest models [53,54]. Piecewise structural equation models (pSEMs) were then applied to characterize direct and indirect correlations of spatial, climatic, soil, and vegetation variables with beetle activity density, α–diversity, and β–diversity. These models assessed the consistency of the data with hypothesized pathways [55,56]. The pSEM were fitted using the same site–level dataset [56]. All continuous environmental variables were Z–score standardized. Principal component analysis (PCA) was performed separately for the spatial, climatic, soil, and vegetation variable sets, and the first principal component (PC1) was retained only when it represented a biologically interpretable proportion of variation [57,58]. The variance explained by each PC1 and the loadings of all contributing variables were reported in a Supplementary Tables S4 and S5. The spatial composite comprised longitude, latitude, and elevation. The climatic composite comprised mean annual temperature, mean annual relative humidity, and mean annual precipitation. Soil Composite 1 comprised total carbon, total nitrogen, and total phosphorus; Soil Composite 2 comprised soil water content and soil temperature; and Soil Composite 3 comprised soil compaction and soil pH. The vegetation composite comprised aboveground biomass, plant density, vegetation cover, plant species richness, and plant height. The pSEMs were implemented using the piecewise SEM package, and all analyses were conducted in R version 4.2.3 [56].

## 3. Results

### 3.1. Taxonomic Composition of Ground–Active Beetle Assemblages

A total of 48,093 ground–active beetles were collected, comprising 105 species, 62 genera, and 10 families. The assemblage was concentrated in Scarabaeidae, Carabidae, and Tenebrionidae, which together accounted for 46,110 individuals (95.9% of the total catch) and 85 species. Scarabaeidae was the most abundant family, with 22,394 individuals representing 23 species, whereas Carabidae had the greatest species richness, with 19,381 individuals representing 41 species. Tenebrionidae contributed a further 4335 individuals and 21 species. The remaining seven families collectively comprised only 1983 individuals and 20 species.

### 3.2. Regional Taxonomic and Feeding–Guild Structure

**Figure 2:**
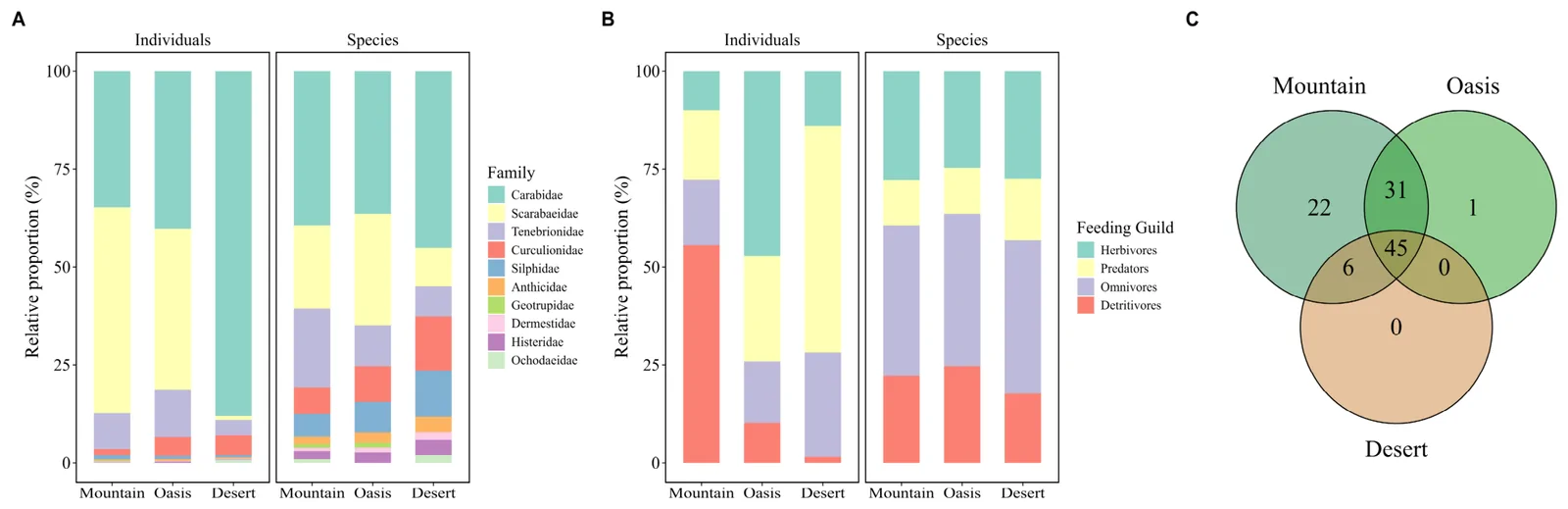
Relative abundances and Venn diagrams of beetle families and trophic groups in surface communities across different regions. (**A**) Relative proportions (%) of beetle individuals and species by family in the mountainous, oasis, and desert regions. (**B**) Relative proportions (%) of beetle individuals and species by feeding guild (herbivores, predators, omnivores, and detritivores) in the three regions. (**C**) Venn diagram showing the number of beetle species unique to and shared among the mountainous, oasis, and desert regions; the overlapping area indicates species shared between two or all three regions.

Ground–active beetle composition differed markedly among regions (Figure 2). Most species and individuals in mountainous and oasis regions belonged to Carabidae, Scarabaeidae, and Tenebrionidae, whereas desert sites were numerically dominated by Carabidae. Across the complete dataset, herbivores comprised 41 species from 28 genera and five families, predators 29 species from 20 genera and two families, omnivores 12 species from seven genera and four families, and detritivores 23 species from nine genera and four families (Table 1, Figure 2A). Feeding–guild dominance differed among regions: detritivores accounted for 55.63% of individuals at mountainous sites, herbivores for 47.18% at irrigated–oasis sites, and predators for 57.65% at desert region (Figure 2B). Forty–five species occurred in all three regions. Twenty–two species were recorded only in the mountainous region, one only in the oasis region, and none only in the desert region (Figure 2C).

### 3.3. Activity Density and Diversity of Ground–Active Beetle Assemblages

**Figure 3:**
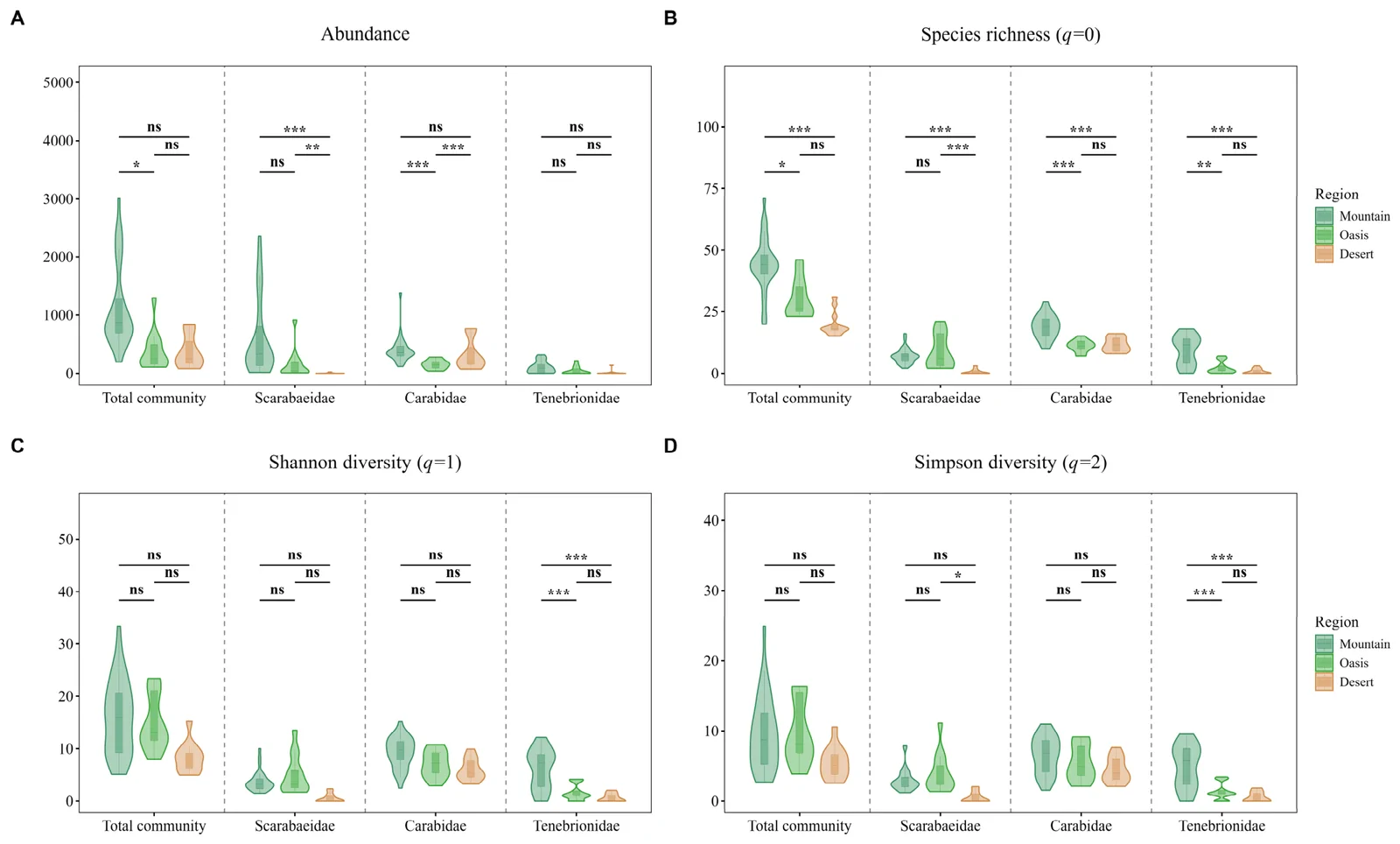
Abundance and α–diversity of the total community and the dominant families. (**A**) Abundance (number of individuals) of the total beetle community and of the dominant families (Scarabaeidae, Carabidae, and Tenebrionidae) in each region. (**B**) Species richness (Hill number, *q* = 0) of the total community and dominant families in each region. (**C**) Shannon diversity (Hill number, *q* = 1) of the total community and dominant families in each region. (**D**) Simpson diversity (Hill number, *q* = 2) of the total community and dominant families in each region. In each panel, violin plots show the distribution of values across sampling fields for the mountainous, oasis, and desert regions; horizontal bars indicate pairwise comparisons, with significance levels denoted as * *p* < 0.05, ** *p* < 0.01, *** *p* < 0.001, and ns = not significant.

**Figure 4:**
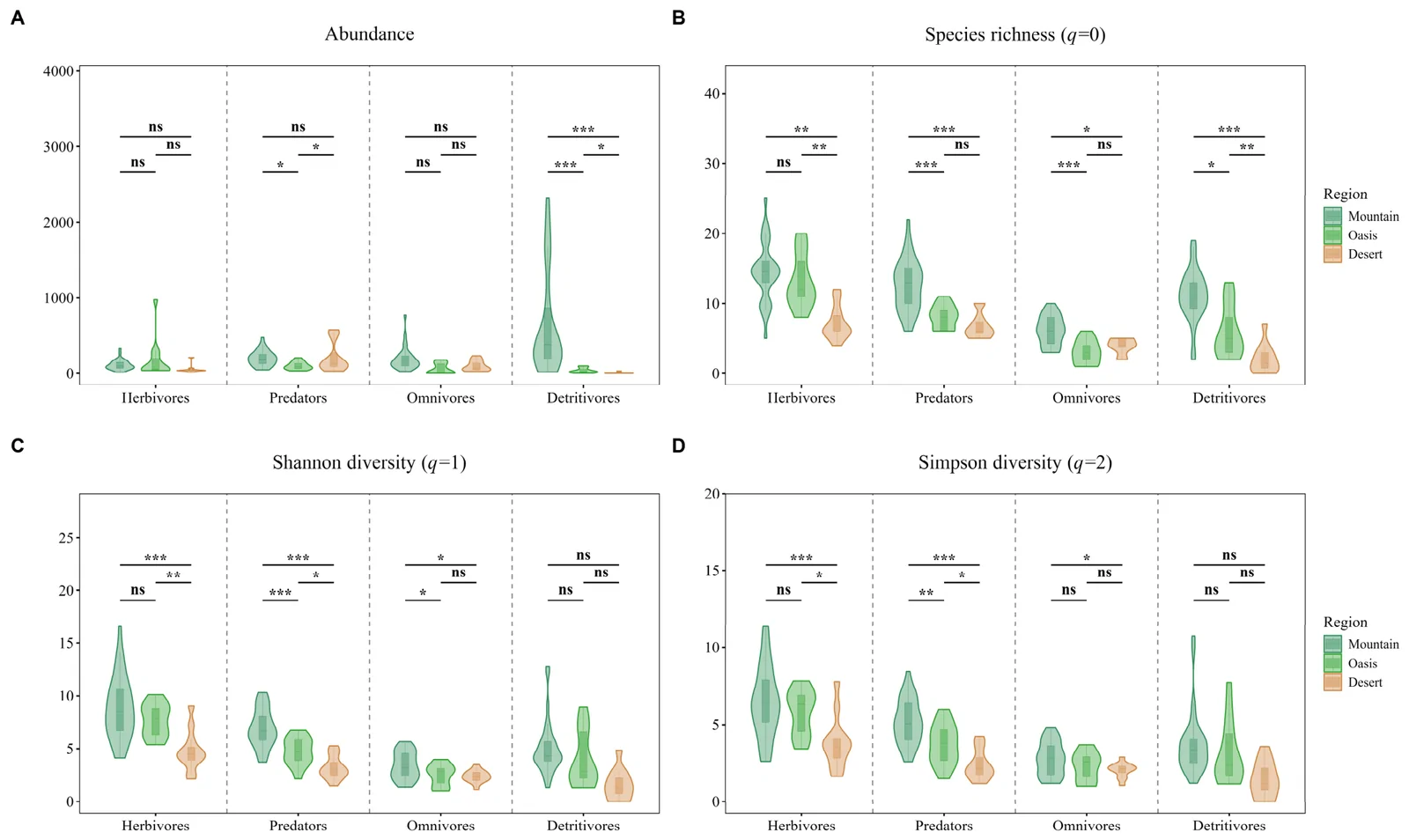
Abundance and α–diversity of different nutritional groups. (**A**) Abundance (number of individuals) of herbivores, predators, omnivores, and detritivores in each region. (**B**) Species richness (Hill number, *q* = 0) of each feeding guild in each region. (**C**) Shannon diversity (Hill number, *q* = 1) of each feeding guild in each region. (**D**) Simpson diversity (Hill number, *q* = 2) of each feeding guild in each region. In each panel, violin plots show the distribution of values across sampling fields for the mountainous, oasis, and desert regions; horizontal bars indicate pairwise comparisons, with significance levels denoted as * *p* < 0.05, ** *p* < 0.01, *** *p* < 0.001, and ns = not significant (*p* ≥ 0.05).

Agroecological region was significantly associated with the activity density and alpha diversity of the total assemblage, dominant families, and several feeding guilds (Figure 3 and Figure 4; Tables S1 and S2). The activity densities of the total assemblage and Carabidae were higher in the mountainous region than in the oasis region, whereas Scarabaeidae activity density was higher in the mountainous region than in the desert region. Species richness of the total assemblage, Carabidae, and Tenebrionidae was greater in the mountainous region than in either the oasis or desert region. Scarabaeidae Shannon diversity was higher in the oasis region than in the desert region, whereas both Shannon and Simpson diversity of Tenebrionidae were higher in the mountainous region than in the other two regions.

Feeding guilds showed distinct regional patterns (Figure 4, Table S2). Predator activity density was higher in the mountainous and desert regions than in the oasis region. Detritivore activity density declined from the mountainous region to the oasis and desert regions, with significant differences among all pairs. The mountainous region also supported greater species richness of predators, omnivores, and detritivores than the other two regions. Herbivore Shannon and Simpson diversity were higher in the mountainous and oasis regions than in the desert region. Predator diversity was highest in the mountainous region, intermediate in the oasis region, and lowest in the desert region. Omnivore Shannon diversity was higher in the mountainous region than in both other regions, whereas omnivore Simpson diversity differed significantly only between the mountainous and desert regions. Whole–assemblage species richness showed clear regional differentiation, whereas Shannon and Simpson diversity did not exhibit corresponding changes.

### 3.4. Community Composition and Beta Diversity

**Figure 5:**
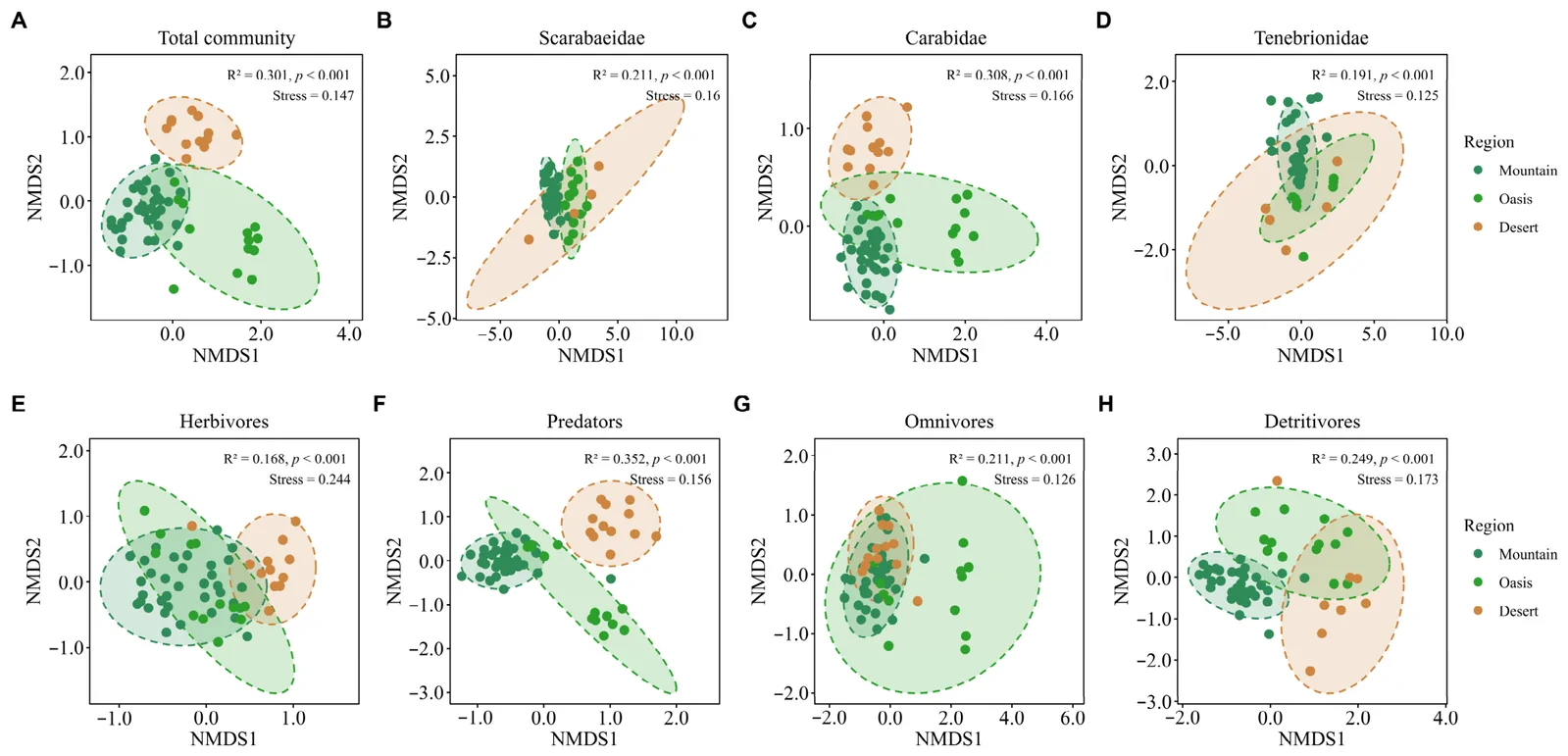
NMDS analysis of the total community, dominant families, and nutritional groups. *R*^2^ and *p*–values are from PERMANOVA tests, and stress values indicate the goodness of fit of each ordination. (**A**) Total community; (**B**) Scarabaeidae; (**C**) Carabidae; (**D**) Tenebrionidae; (**E**) Herbivores; (**F**) Predators; (**G**) Omnivores; (**H**) Detritivores.

NMDS ordinations showed clear regional differentiation in the composition of the total ground–active beetle assemblage, Scarabaeidae, Carabidae, Tenebrionidae, and all four feeding guilds (Figure 5). PERMANOVA confirmed these differences. Agroecological region explained 30.1% of compositional variation in the total assemblage (R^2^ = 0.301, *p* < 0.001) and 35.2% of compositional variation in the predatory guild. Most pairwise regional contrasts were significant, with the exception of Scarabaeidae and Tenebrionidae between the oasis and desert regions (Table S3).

**Figure 6:**
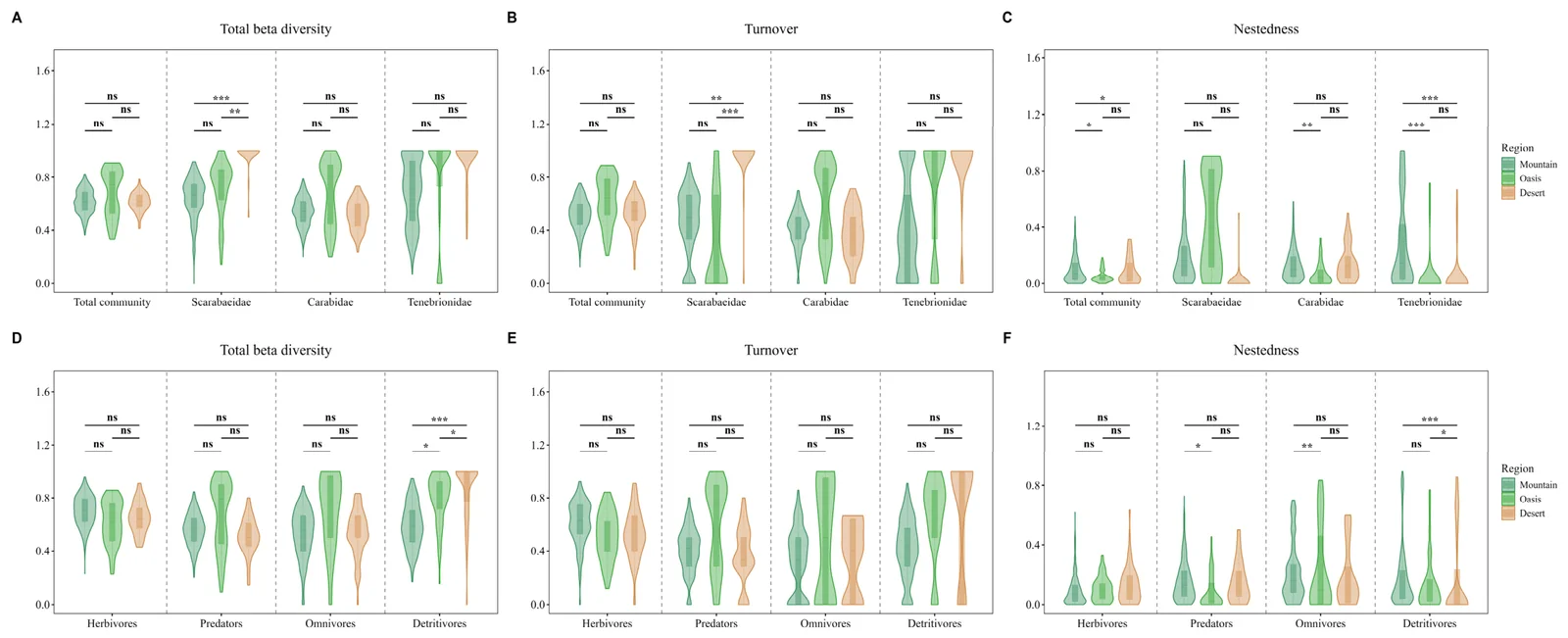
Beta diversity of the total community, dominant families, and nutritional groups. (**A**) Total beta diversity of community; (**B**) Turnover component of community; (**C**) Nestedness components of community; (**D**) Total beta diversity of feeding guilds; (**E**) Turnover component of feeding guilds; (**F**) Nestedness component of feeding guilds. Significance levels: ns, *p* > 0.05; * *p* < 0.05; ** *p* < 0.01; *** *p* < 0.001.

Beta diversity partitioning indicated that the form of regional differentiation varied among taxonomic groups. For the total assemblage, Carabidae, and Tenebrionidae, significant regional differences were concentrated in the nestedness component. The mountainous region had higher nestedness for the total assemblage and Tenebrionidae than the oasis and desert regions, whereas mountainous Carabidae nestedness was higher than that of the oasis region. Scarabaeidae instead showed significant regional differences in total beta diversity and turnover, with mountainous and oasis values higher than those of the desert region (Figure 6A–F; Tables S4 and S5).

### 3.5. Environmental Correlates of Activity Density and Alpha Diversity

**Figure 7:**
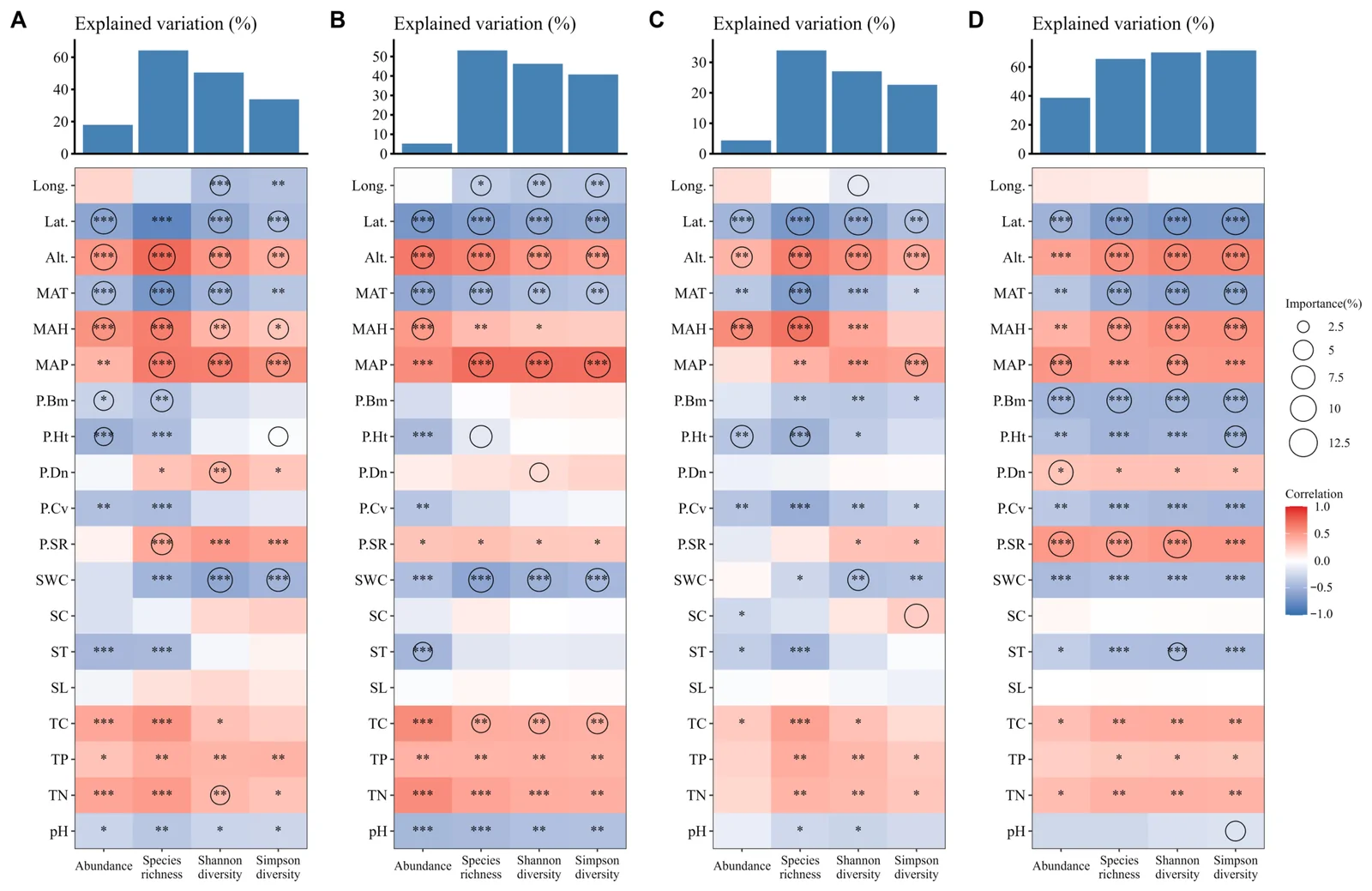
Heatmap and random forest analysis of correlations between total community, dominant family α–diversity, and abundance with environmental factors. (**A**) Total community; (**B**) Scarabaeidae; (**C**) Carabidae; (**D**) Tenebrionidae. Significance levels: * *p* < 0.05; ** *p* < 0.01; *** *p* < 0.001. For the abbreviations, see Section 2.

Spearman correlations described the direction of bivariate associations, and random forest models ranked the relative importance of environmental predictors (Figure 7). For the total assemblage, elevation, latitude, MAP, MAT, and MAH were the principal predictors. Predictor rankings differed among dominant families. Scarabaeidae were most strongly associated with elevation, latitude, MAP, soil compaction, and soil organic matter, whereas Carabidae were primarily associated with elevation and latitude. Tenebrionidae were associated with a broader set of variables, including latitude, elevation, plant biomass, MAT, MAH, and plant species richness.

**Figure 8:**
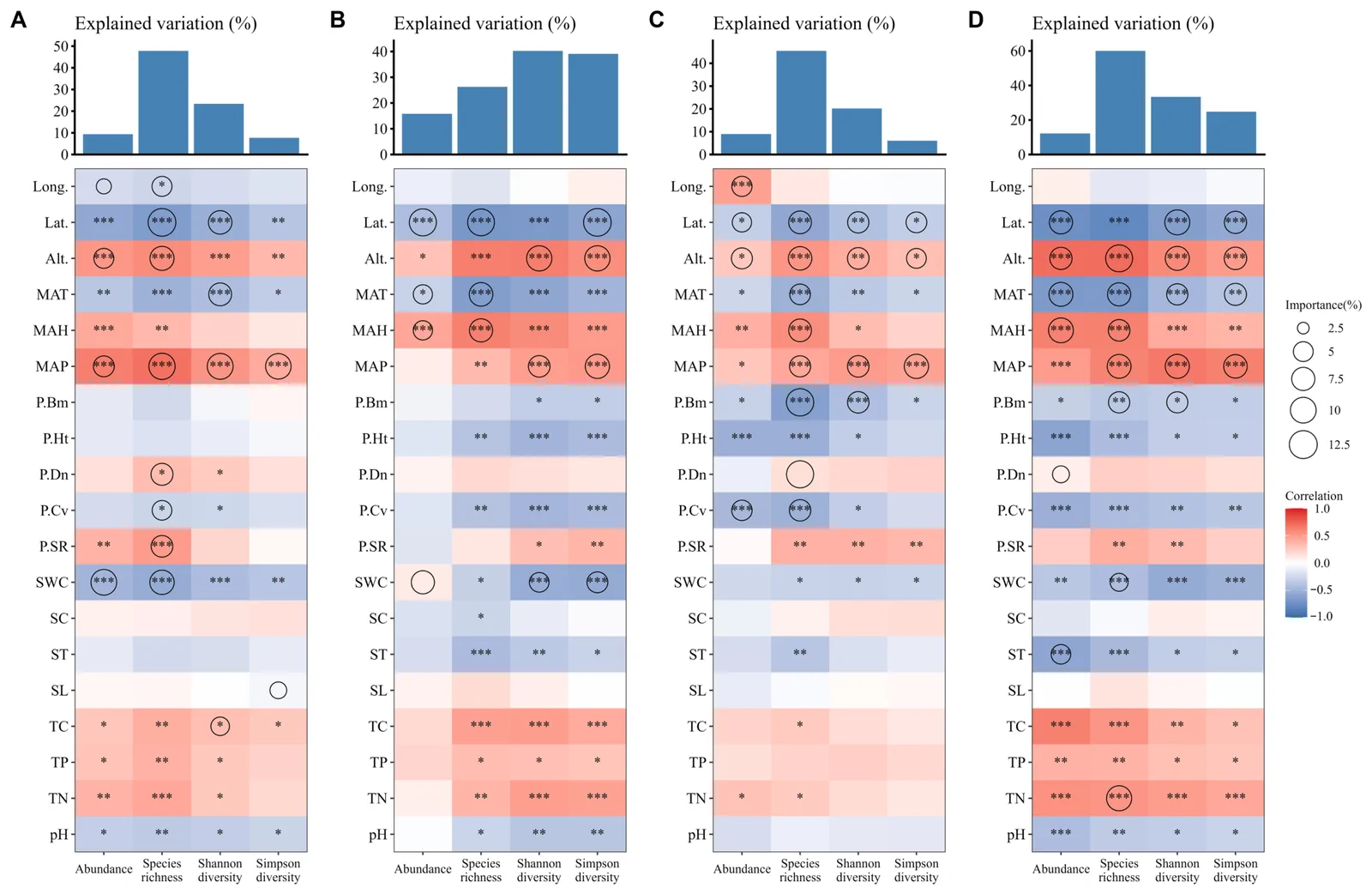
Heatmap and random forest analysis of correlations between α–diversity and abundance of different nutritional groups and environmental factors. (**A**) Herbivores; (**B**) predators; (**C**) omnivores; (**D**) detritivores. Significance levels: * *p* < 0.05; ** *p* < 0.01; *** *p* < 0.001.For the abbreviations, see the Section 2.

Environmental associations also differed among feeding guilds (Figure 8). Herbivores were principally associated with MAP, soil compaction, elevation, and latitude. Predator activity density and alpha diversity were most strongly associated with elevation, latitude, MAT, and MAH. Latitude, elevation, MAP, and plant biomass were the principal predictors for omnivores. Detritivore responses were associated with elevation, latitude, MAT, MAP, MAH, and plant biomass.

### 3.6. Direct and Indirect Environmental Associations

**Figure 9:**
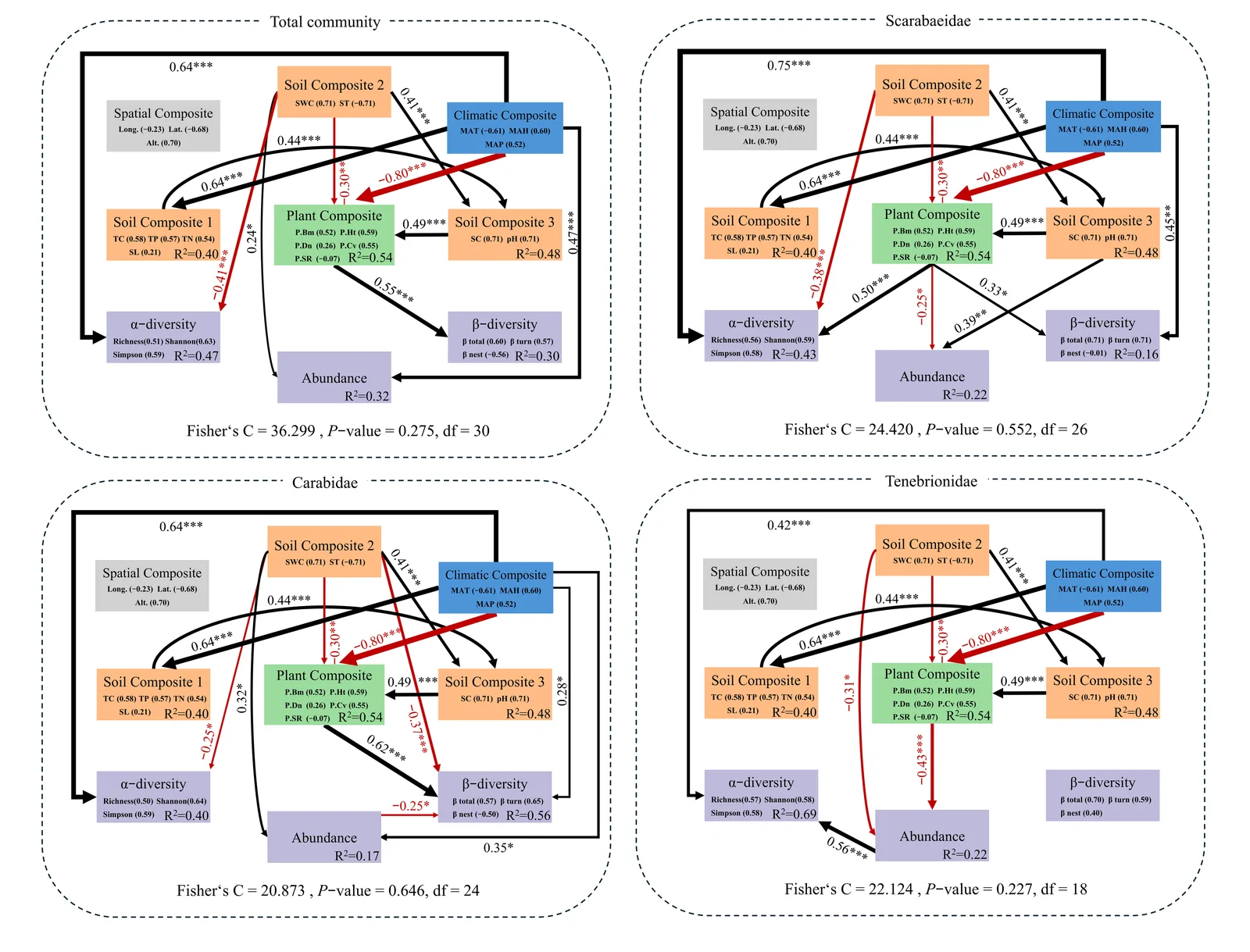
Structural equation model (SEM) shows correlational relationships between environmental factors and the abundance and diversity of the total community and dominant beetle families. *R*^2^ values represent the proportion of variance explained for each dependent variable in the model. Numbers indicate standardized path coefficients, with arrows proportional to the strength of each relationship. Black arrows denote positive correlations, red arrows denote negative correlations, and light–gray dotted arrows indicate non–significant relationships (*p* > 0.05). Significance levels are indicated by *, **, and *** for *p* < 0.05, *p* < 0.01 and *p* < 0.001, respectively.

**Figure 10:**
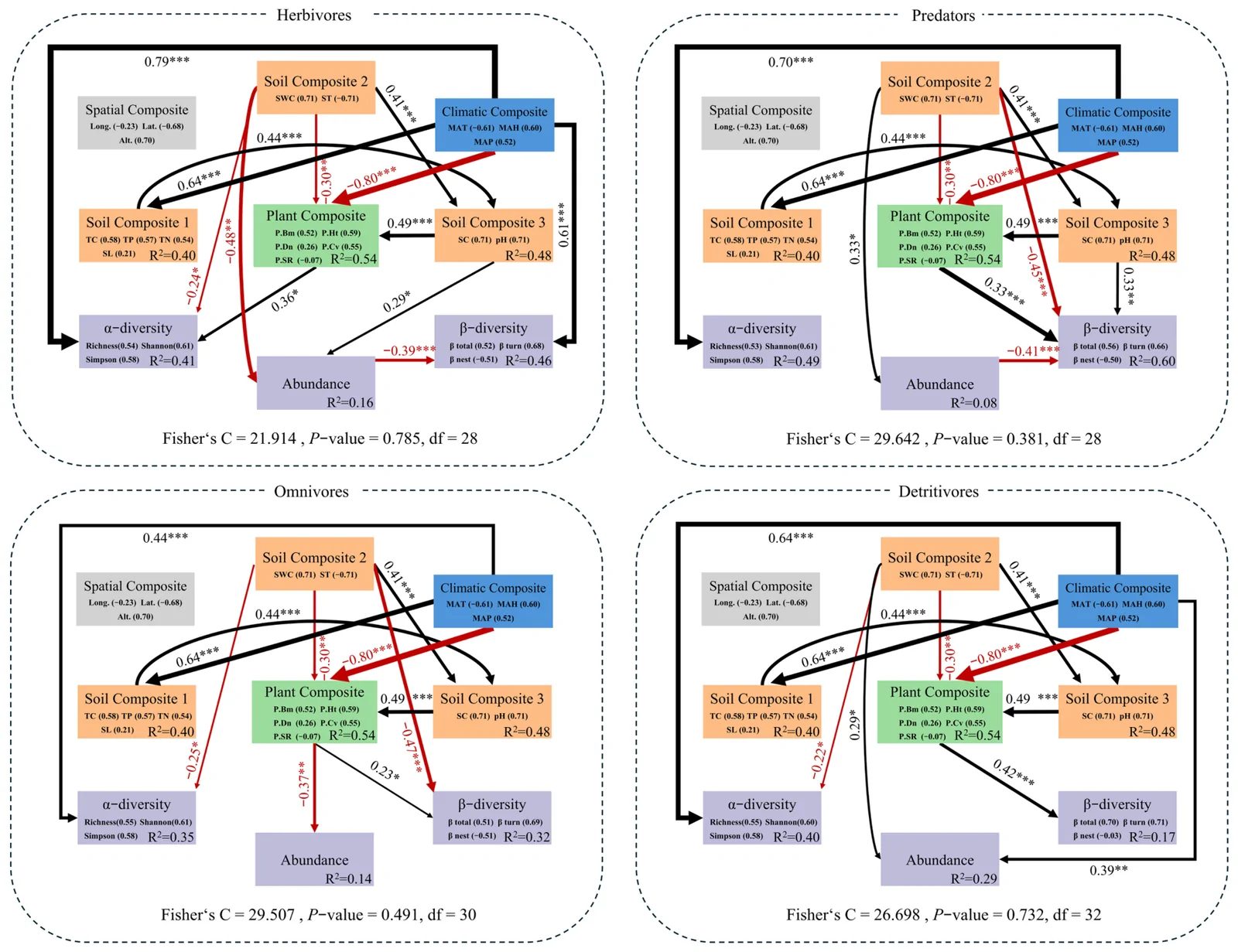
Structural equation model (SEM) showing correlational relationships between environmental factors and the abundance and diversity of different trophic guilds. *R*^2^ values represent the proportion of variance explained for each dependent variable in the model. Numbers indicate standardized path coefficients, with arrow width proportional to the strength of the relationship. Black arrows represent positive coefficients, red arrows represent negative coefficients, and dotted arrows in light gray indicate no significant relationship (*p* > 0.05). Significance levels are indicated by *, **, and *** for *p* < 0.05, *p* < 0.01 and *p* < 0.001, respectively.

The pSEMs identified group–specific direct and indirect associations among climate, soil, vegetation, activity density, alpha diversity, and beta diversity (Figure 9 and Figure 10). For the total assemblage, activity density was positively associated with the climatic composite and Soil Composite 2. Alpha diversity was positively associated with the climatic composite and negatively associated with Soil Composite 2, whereas beta diversity was positively associated only with the vegetation composite.

Responses differed among dominant families. Scarabaeidae activity density was positively associated with Soil Composite 3 and negatively associated with the vegetation composite. Scarabaeidae alpha diversity was positively associated with the climatic and vegetation composites and negatively associated with Soil Composite 2; its beta diversity was positively associated with the climatic and vegetation composites. Carabidae activity density was positively associated with the climatic composite and Soil Composite 2. Carabidae alpha diversity was positively associated with the climatic composite and negatively associated with Soil Composite 2, whereas its beta diversity was positively associated with the climatic and vegetation composites and negatively associated with Soil Composite 2 and activity density. Tenebrionidae activity density was negatively associated with Soil Composite 2 and the vegetation composite, while its alpha diversity was positively associated with the climatic composite and activity density (Figure 9).

Guild–level models showed a similar hierarchy but distinct direct correlational links (Figure 10). Herbivore activity density was negatively associated with Soil Composite 2 and positively associated with Soil Composite 3. Herbivore alpha diversity was positively associated with the climatic and vegetation composites, while beta diversity was positively associated with climate and negatively associated with activity density. Predator activity density was positively associated with Soil Composite 2, predator alpha diversity with the climatic composite, and predator beta diversity positively with the vegetation composite and Soil Composite 3 but negatively with Soil Composite 2 and activity density. Omnivore activity density was negatively associated with the vegetation composite; omnivore alpha diversity was positively associated with climate and negatively with Soil Composite 2, whereas beta diversity was positively associated with vegetation and negatively associated with Soil Composite 2. Detritivore activity density was positively associated with the climatic composite and Soil Composite 2; detritivore alpha diversity was positively associated with climate and negatively associated with Soil Composite 2, whereas beta diversity was positively associated only with the vegetation composite.

## 4. Discussion

This study indicated that regional differentiation in ground–active beetle assemblages remains detectable within perennial alfalfa fields. Mountainous regions supported the largest observed species pool and were dominated by detritivores; Oasis regions had a higher relative abundance of herbivores, but herbivore activity density was not higher. Desert sites were numerically dominated by carabids and predators but had relatively low predator richness and diversity. For the total assemblage, regional differences in beta diversity primarily involved the nestedness–resulant component, whereas family–level patterns also included turnover. In the fitted models, climate was associated with beetle responses both directly and indirectly through pathways involving soil and vegetation. The direction and strength of these associations varied among families, feeding guilds, and response dimensions. Together, these findings show that cultivation of the same crop does not imply ecological equivalence among dryland regions. They also suggest that a single taxonomic group or diversity metric may not fully capture regional variation in assemblage structure.

### 4.1. Regional Differentiation Persisted Within Alfalfa Fields

Perennial alfalfa fields are characterized by relatively continuous vegetation cover, a relatively stable near–surface microclimate, and limited tillage disturbance [17,18,19]. Despite this shared habitat template, agroecological region explained 30.1% of the variation in overall beetle assemblage composition and 35.2% of the variation in predators’ assemblage composition. These results suggest that crop identity alone is insufficient to homogenize beetle communities across regions. Instead, regional variation in elevation, hydrothermal conditions, soil properties, irrigation, and surrounding landscape features may contribute to the observed assemblage differences. This pattern is consistent with evidence that farmland biodiversity depends on habitat heterogeneity and landscape context across multiple spatial scales [23,25,27]. It suggests that regional environmental differences remain relevant even where the same perennial crop is cultivated. However, the data support an association between assemblage composition and regional context as a whole. They do not allow the independent contributions of elevation, aridity, or irrigation to be isolated.

### 4.2. Taxonomic and Feeding–Guild Components Were Reorganized Non–Congruently

Mountainous sites supported greater overall species richness as well as higher richness within Carabidae and Tenebrionidae, and within the predators, omnivores, and detritivores. These patterns are consistent with the cooler, wetter, and more heterogeneous conditions at mountainous sites [26]. Higher soil moisture can promote surface activity of ground–active beetles [59], while plant diversity and structural complexity provide shelter, buffer microclimatic fluctuations, and support persistence or recolonization [60]. Variation in carabid responses to soil management further suggests that soil pore structure and the availability of organic resources influence habitat suitability for different ecological groups [61]. These processes may help explain the numerical dominance and comparatively high species richness of detritivores in the mountainous region, where they accounted for 55.63% of captured individuals [59,60,61]. Nevertheless, near–surface temperature and humidity, litter quality, dung availability, and prey abundance were not measured directly. The proposed pathway involving resources and microclimate therefore remains a plausible interpretation of the observed associations, not a confirmed causal mechanism.

Herbivores accounted for 47.18% of individuals in the oasis region, yet their activity density did not differ significantly among regions, and their richness and diversity were not highest in the oasis region. This apparent contrast underscores the distinction between relative abundance and activity. A high herbivore proportion indicates a larger share of the local assemblage, but does not necessarily imply greater absolute abundance or a higher risk of crop damage. Irrigation may extend the period of alfalfa resource availability and alleviate seasonal water limitation. However, ground–active beetles often respond nonlinearly to resource gradients and agricultural disturbance [26]. The oasis pattern is consistent with a shift in feeding–guild composition that may reflect resource subsidies, mowing disturbance, and changes in the abundance of other guilds [17,19]. Direct measurements of herbivory, plant injury, and yield loss would be needed to assess whether this compositional pattern indicates increased pest risk.

Carabids and predators accounted for most captured individuals in the desert region, where predator richness and diversity were comparatively low. This pattern suggests that taxa may respond differently to harsh conditions, potentially favoring a few tolerant or highly mobile species. Agricultural landscape simplification can selectively filter habitat specialists and dispersal–limited species even when aggregate richness changes little [62]. Beetle responses to cropland and landscape structure can also vary with feeding guild and dispersal traits [13,62]. However, the high proportion of predators at desert sites should not be interpreted as evidence of stronger biological control. Pitfall traps measure activity density rather than absolute abundance, and capture rates are influenced by mobility, surface temperature, and vegetation structure [28,29,30].

### 4.3. α– and β–Diversity Revealed Complementary Dimensions of Community Change

Agroecological region was associated with whole–assemblage species richness but not with Shannon or Simpson diversity. These results are complementary because the metrics differ in their sensitivity to species richness and distribution of relative abundances [41,42,43]. The larger species pool and greater number of region–specific taxa in the mountainous region may not have been accompanied by changes in relative abundance sufficient to produce detectable differences in Shannon or Simpson diversity. At the family and feeding–guild levels, Shannon and Simpson diversity showed significant regional differences, but the patterns varied among groups. These contrasting responses suggest that whole–assemblage indices may obscure differences among families and feeding guilds. This interpretation is consistent with evidence that community change is not always reflected in species–richness trends [63].

For the total assemblage, regional differences in *β*–diversity primarily involved the nestedness–resultant component. Forty–five species were shared across regions, whereas 22 were recorded only in the mountainous region, one only in the oasis region, and none exclusively in the desert region. These patterns are consistent with species–poor assemblages forming subsets of richer assemblages [50,51]. In contrast to the turnover–dominated pattern often observed along environmental gradients [64,65], the regional differences in the nestedness–resultant component may be consistent with species–poor assemblages forming subsets of richer assemblages under greater water limitation within a shared crop habitat. However, this interpretation does not apply uniformly across families: Scarabaeidae showed regional differences in both total *β*–diversity and turnover, in addition to the broader pattern involving nestedness. Nestedness alone does not establish environmental filtering as the underlying mechanism. Unequal sample sizes among regions may limit the strength of regional comparisons. The large number of mountainous sites, together with regional differences in sampling coverage, detection probability, spatial extent, and species pools, may affect estimates of species richness, the number of region species, and β–diversity. Coverage–based rarefaction, null models, and spatially explicit analyses would complement balanced resampling [52] in evaluating possible explanations for the observed species–subset pattern [66,67,68]. PERMANOVA results should likewise be evaluated alongside tests of homogeneity of multivariate dispersion [48,49]. Spatial autocorrelation was not formally assessed. Geographic clustering of sites within regions may affect inference from PERMANOVA and correlation analyses. Because geographic location covaried with regional environmental conditions, the respective contributions of spatial proximity and environmental differences remain unresolved. The observed pattern is consistent with species–poor assemblages potentially through regional environmental filtering. However, the data do not establish the underlying mechanism.

### 4.4. Environment Was Associated with Different Response Dimensions Through Group–Specific Pathways

Spearman correlation analyses, random forests models and piecewise structural equation models (pSEMs) provided complementary perspectives on the same observational dataset [55,56]. Environmental associations varied among beetle groups. In the fitted pSEMs, climatic variables were associated with activity density and *α*–diversity through both direct and indirect pathways involving local soil and vegetation conditions. Regional identity covaried with climate, soils, and irrigation and may also have covaried with unmeasured management practices. The pSEM path coefficients should be interpreted as correlational associations conditional on the assumed causal structure, not as demonstrated causal effects [55,58]. This limitation should guide the interpretation of group–specific patterns.

Environmental associations also differed among response dimensions [8,27]. Activity density and alpha diversity were primarily associated with climatic gradients, whereas beta diversity and assemblage composition were more consistently associated with vegetation. This pattern is consistent with a possible role for vegetation in species sorting. Differences among response dimensions were accompanied by contrasting patterns among Scarabaeidae, Carabidae, Tenebrionidae, and detritivores, herbivores, and predators. Together, these findings caution against using Carabidae as a universal proxy for the entire ground–active beetle assemblage [25,62]. These differences may reflect variation in drought tolerance and dispersal capacity, traits that are particularly relevant in dryland systems [8,13]. However, neither trait was directly measured in this study. More broadly, these findings imply that environmental correlates in farmland beetles depend on both the taxonomic group or feeding guild examined and the response measured, such as activity density, local richness, or assemblage composition. Monitoring and experimental design that rely on a single metric may therefore fail to capture the full range of biodiversity responses in agroecosystems.

### 4.5. Study Limitations, Management Implications, and Future Directions

Interpretation is constrained by unequal sampling effort and spatial coverage, potential spatial dependence among sites, and covariation of regional identity with climate, soil, irrigation, and potentially unmeasured management practices. Additional limitations include pitfall–trap bias toward surface–active taxa, trophic assignments based on genus– or family–level information, and the absence of direct measurements of predation, decomposition, herbivory, and yield. Nevertheless, our results suggest priorities for region–specific management research: testing the benefits of maintaining habitat heterogeneity in mountainous areas, determining whether herbivore dominance corresponds to crop damage in oases, and evaluating the effects of reducing soil compaction while retaining surface cover at desert–edge sites. These priorities could be investigated using balanced designs with spatial replication, occupancy and null models [66,67,68], and experiments manipulating moisture, litter, vegetation cover, and refuge strips. Functional–trait or molecular dietary analyses [69,70] could further clarify links between assemblage composition and agroecosystem services.

## 5. Conclusions

Ground–active beetle assemblages differed markedly among alfalfa fields in mountainous, irrigated–oasis, and desert–edge regions despite cultivation of the same perennial crop. Regional differences were most evident in species richness, taxonomic composition, feeding–guild organization, and beta diversity components. Whole–assemblage Shannon and Simpson diversity did not show corresponding regional patterns. Mountainous sites supported the largest observed species pool and assemblages dominated by detritivores. Irrigated–oasis sites had a higher proportional contribution of herbivores, but herbivore activity density was not higher. Predators dominated catches at desert sites despite their relatively low species richness. For the total assemblage, regional differences in beta diversity primarily involved the nestedness–resultant component, whereas family–level patterns also involved turnover. In the fitted models, climatic conditions were associated with beetle responses through direct pathways and indirect pathways involving soil and vegetation. These associations varied among taxonomic groups, feeding guilds, and response dimensions. These findings highlight the importance of regional context for biodiversity in dryland farmland. Cultivation of the same crop does not imply ecological equivalence, and a single taxonomic group or diversity metric may not fully capture regional variation in beetle assemblages.

## Figures and Tables

**Table 1:** Taxonomic composition of the ground–active beetle assemblage across all alfalfa sites.

Family	Species (*n*)	Individuals (*n*)
Carabidae	41	19,381
Geotrupidae	1	94
Ochodaeidae	1	57
Scarabaeidae	23	22,394
Tenebrionidae	21	4335
Histeridae	2	75
Silphidae	6	465
Dermestidae	1	82
Curculionidae	7	1073
Anthicidae	2	137
Total	105	48,093

## Data Availability

The original contributions presented in this study are included in the article/Supplementary Materials. Further inquiries can be directed to the corresponding authors.

## Supplementary Materials

The supporting information can be downloaded at https://www.mdpi.com/article/10.3390/insects17101030/s1.
