# Supplementary material for: Regional Differentiation of Ground–Active Beetle Assemblages Across Contrasting Dryland Alfalfa Agroecosystems
Source: Insects. 2026 Oct 8;17(10):1030. doi: 10.3390/insects17101030 (PMC13705272; doi:10.3390/insects17101030)
Supplement: Supplementary file 1 [file insects-17-01030-s001.zip › insects-4553175-supplementary.pdf]

Table S1 Generalized linear mixed-model tests of habitat effects on activity-density and alpha diversity of the total assemblage and dominant beetle families. Values are chi-square statistics with P values in parentheses.

Community metrics	*df*	Total community				Dominant family											
						Scarabaeidae				Carabidae				Tenebrionidae			
		χ^2 value	*P* value	BH-adjusted	FDR verdict	χ^2 value	*P* value	BH-adjusted	FDR verdict	χ^2 value	*P* value	BH-adjusted	FDR verdict	χ^2 value	*P* value	BH-adjusted	FDR verdict
Abundance	2	9.399	0.009**	0.018	Significant	25.549	<0.001***	0.0025	Significant	29.951	<0.001***	0.0025	Significant	4.011	0.135	0.1989	ns
Species richness	2	20.992	<0.001***	0.0025	Significant	15.741	<0.001***	0.0025	Significant	52.255	<0.001***	0.0025	Significant	21.242	<0.001***	0.0025	Significant
Shannon diversity	2	3.631	0.163	0.2341	ns	4.980	0.0829	0.1255	ns	6.139	0.046*	0.0812	ns	43.865	<0.001***	0.0025	Significant
Simpson diversity	2	2.506	0.286	0.3559	ns	6.140	0.0464*	0.082	ns	2.188	0.335	0.4078	ns	42.217	<0.001***	0.0025	Significant

Table S2. Generalized linear mixed-model tests of habitat effects on activity-density and alpha diversity of trophic guilds. Values are chi-square statistics with P values in parentheses.

Community metrics	*df*	Feeding guild															
		Herbivores				Predators				Omnivores				Detritivores			
		χ^2 value	*P* value	BH-adjusted	FDR verdict	χ^2 value	*P* value	BH-adjusted	FDR verdict	χ^2 value	*P* value	BH-adjusted	FDR verdict	χ^2 value	*P* value	BH-adjusted	FDR verdict
Abundance	2	1.560	0.458	0.053	ns	10.069	0.007**	0.014	Significant	3.532	0.171	0.239	ns	55.032	<0.001***	0.002	Significant
Species richness	2	12.970	0.002**	0.0047	Significant	31.293	<0.001***	0.002	Significant	20.282	<0.001***	0.003	Significant	25.042	<0.001***	0.002	Significant
Shannon diversity	2	24.603	<0.001***	0.003	Significant	58.018	<0.001***	0.002	Significant	11.969	0.003**	0.006	Significant	5.653	0.059	0.09	ns
Simpson diversity	2	15.546	<0.001***	0.002	Significant	42.899	<0.001***	0.003	Significant	6.779	0.034*	0.06	ns	5.342	0.069	0.06	ns

Table S3. Pairwise PERMANOVA comparisons of community composition among habitat settings.

Assemblage component	Comparison	F	R^2	*P*
Total assemblage	Mountain vs oasis	11.247	0.199	<0.001
Total assemblage	Mountain vs desert	14.644	0.250	<0.001
Total assemblage	Oasis vs desert	9.289	0.288	<0.001
Scarabaeidae	Mountain vs oasis	9.691	0.177	<0.001
Scarabaeidae	Mountain vs desert	4.826	0.118	<0.001
Scarabaeidae	Oasis vs desert	1.855	0.110	0.096
Carabidae	Mountain vs oasis	12.313	0.215	<0.001
Carabidae	Mountain vs desert	14.775	0.251	<0.001
Carabidae	Oasis vs desert	9.383	0.289	<0.001
Tenebrionidae	Mountain vs oasis	6.298	0.139	<0.001
Tenebrionidae	Mountain vs desert	5.348	0.136	<0.001
Tenebrionidae	Oasis vs desert margin	1.618	0.111	0.510
Herbivores	Mountain vs oasis	4.182	0.085	<0.001
Herbivores	Mountain vs desert	7.043	0.138	<0.001
Herbivores	Oasis vs desert	5.945	0.205	<0.001
Predators	Mountain vs oasis	12.075	0.212	<0.001
Predators	Mountain vs desert	21.914	0.332	<0.001
Predators	Oasis vs desert	10.446	0.312	<0.001
Omnivores	Mountain vs oasis	10.068	0.183	<0.001
Omnivores	Mountain vs desert	4.212	0.087	0.006
Omnivores	Oasis vs desert	7.221	0.239	0.002
Detritivores	Mountain vs oasis	10.008	0.182	<0.001
Detritivores	Mountain vs desert	10.659	0.206	<0.001
Detritivores	Oasis vs desert	3.608	0.153	0.002

Table S4 Results of testing the effects of site type, year, and their interaction on the β diversity of total ground beetle communities and dominant families, as well as their decomposition components, based on generalized linear mixed models (GLMM)

Index	*df*	Total assemblage				Dominant families											
						Scarabaeidae				Carabidae				Tenebrionidae			
		χ^2 value	*P* value	BH-adjusted	FDR verdict	χ^2 value	*P* value	BH-adjusted	FDR verdict	χ^2 value	*P* value	BH-adjusted	FDR verdict	χ^2 value	*P* value	BH-adjusted	FDR verdict
Total beta-diversity	2	0.036	0.982	0.98	ns	15.878	<0.001***	0.002	Significant	0.666	0.717	0.76	ns	2.813	0.245	0.31	ns
Turn over	2	0.272	0.873	0.88	ns	14.070	<0.001***	0.002	Significant	1.165	0.559	0.62	ns	3.417	0.181	0.24	ns
Nestedness	2	12.668	0.002**	0.004	Significant	5.577	0.0615	0.09	ns	11.546	0.003**	0.005	Significant	75.426	<0.001***	0.002	Significant

Table S5 Results of testing the effects of site type, year, and their interaction on the β-diversity of different nutritional groups of ground beetles and their decomposition components based on generalized linear mixed models (GLMM)

Index	*df*	Nutritional groups															
		Herbivores				Predators				Omnivores				Detritivores			
		χ^2 value	*P* value	BH-adjusted	FDR verdict	χ^2 value	*P* value	BH-adjusted	FDR verdict	χ^2 value	*P* value	BH-adjusted	FDR verdict	χ^2 value	*P* value	BH-adjusted	FDR verdict
Total beta-diversity	2	3.114	0.211	0.27	ns	0.515	0.7728	0.77	ns	1.523	0.467	0.46	ns	19.974	<0.001***	<0.001	Significant
Turn over	2	3.085	0.214	0.21	ns	0.637	0.727	0.72	ns	0.699	0.705	0.71	ns	5.571	0.062	0.06	ns
Nestedness	2	1.511	0.469	0.46	ns	7.319	0.026*	0.02	Significant	14.331	<0.001***	0.02	Significant	27.856	<0.001***	0.02	Significant

Table S6 The species composition and proportion of ground beetles in different regions

Family	Genus	Species	Individular	Guilds	Reference
Carabidae	*Archastes*	*Archastes solitarius*	20	Predator	[31, 32]
	Carabus	*Carabus brandti*	712	Predator	
		Carabus glyptopterus	62	Predator	
		Carabus vladimirskyi	307	Predator	
	Loricera	*Loricera pilicornis*	22	Predator	
	Calosoma	*Calosoma lugens*	257	Predator	
		Calosoma chinense	38	Predator	
	Scarites	*Scarites terricola*	119	Predator	
	Broscus	*Broscus kozlovi*	1898	Predator	
	Archipatrobus	*Patrobus flavipes*	11	Predator	
	Chlaenius	*Chlaenius pallipes*	272	Predator	
		Chlaenius posticalis	48	Predator	
		Chlaenius naeviger	467	Predator	
	Corsyra	*Corsyra fusula*	175	Predator	
	Harpalus	*Harpalus amplicollis*	2217	Omnivore	
		Harpalus macronotus	571	Omnivore	
		Harpalus capito	878	Predator	
		Harpalus corporosus	270	Herbivore	
		Harpalus calceatus	449	Herbivore	
		Harpalus pallidipennis	530	Predator	
		Harpalus optabilis	108	Herbivore	
		Harpalus salinus	249	Herbivore	
		Harpalus davidi	58	Omnivore	
		Harpalus froelichi	101	Omnivore	
		Harpalus lumbaris	225	Predator	
		Harpalus griseus	115	Herbivore	
		Harpalus sinicus	55	Herbivore	
		Harpalus bungi	4065	Omnivore	
	Cymindis	*Cymindis daimio*	86	Predator	
		Cymindis binotata	55	Predator	
	Martyr	*Martyr alter*	29	Predator	
	Agonum	*Agonum nitidum*	1815	Predator	
	Pterostichus	*Pterostichus gebleri*	278	Predator	
	Dolichus	*Dolichus halensis*	212	Predator	
	Pseudotaphoxenus	*Pseudotaphoxnus mongolicus*	1009	Predator	
		Pseudotaphoxnus brevipennis	954	Predator	
	Reflexisphodrus	*Reflexisphodrus refleximargo*	40	Predator	
	Amara	*Amara banghaasi*	293	Herbivore	
		Amara brevicollis	44	Herbivore	
		Amara dux	70	Herbivore	
	Hexagonia	*Hexagonia*	197	Predator	
Scarabaeidae	*Aphodius*	*Aphodius impunctatus*	69	Detritivore	[33—36]
		Aphodius rectus	3522	Detritivore	
	Popillia	*Popillia quadriguttata*	17	Herbivore	
	Anomala	*Anomala sulcipennis*	55	Herbivore	
		Anomala exoleta	38	Herbivore	
	Chioneosoma	*Chioneosoma reitteri*	26	Herbivore	

		Maladera verticalis	583	Herbivore	
	Maladera				
		Maladera orientalis	287	Herbivore	
	Trematodes	*Trematodes tenebrioides*	224	Herbivore	
	Holotrichia	*Holotrichia oblita*	294	Herbivore	
		Protaetia brevitarsis	1125	Herbivore	
	Protaetia				
		Protaetia famelica	35	Herbivore	
	Cetonia	*Cetonia magnifica*	24	Herbivore	
	Anthracophora	*Anthracophora rusticola*	14	Herbivore	
	Gymnopleurus	*Gymnopleurus mopsus*	787	Detritivore	
		Onthophagus gibbulus	1460	Detritivore	
		Onthophagus bivertex	126	Detritivore	
		Onthophagus marginalis	136	Detritivore	
	Onthophagus	*Onthophagus olsoufieffi*	584	Detritivore	
		Onthophagus sinicus	4552	Detritivore	
		Onthophagus tragus	7705	Detritivore	
		Liatongus phanaeoides	671	Detritivore	
	Scarabaeus	*Scarabaeus typhon*	60	Detritivore	
		Blaps variolosa	226	Detritivore	
		Blaps davidea	672	Detritivore	
	Blaps	*Blaps femoralis*	480	Detritivore	
		Blaps kiritshenkoi	327	Detritivore	
		Blaps variolaris	182	Detritivore	
		Prosodes pekinensis	15	Herbivore	
	Prosodes				
		Prosodes rugulosa	164	Herbivore	
	Platyscelis	*Platyscelis hauseri*	41	Herbivore	
	Scleropatrum	*Scleropatrum horridum*	77	Herbivore	
	Crypticus	*Crypticus rufipes*	334	Herbivore	
Tenebrionidae	*Microdera*	*Microdera kraatzi*	840	Omnivore	[37, 38]
	Gonocephalum	*Gonocephalum reticulatum*	239	Herbivore	
	Opatrum	*Opatrum subaratum*	161	Herbivore	
	Scytosoma	*Scytosoma pygmaeum*	273	Omnivore	
		Anatolica amoenula	29	Omnivore	
	Anatolica				
		Anatolica gravidula	51	Omnivore	
	Sternoplax	*Sternoplax szechenyi*	13	Herbivore	
	Cyphogenia	*Cyphogenia chinensis*	62	Herbivore	
	Eumylada	*Eumylada oberbergeri*	30	Herbivore	
	Penthicus	*Penthicus beicki*	86	Herbivore	
	Platyope	*Platyope mongolica*	33	Herbivore	
